# Research topics and hotspot trends of lumbar spondylolisthesis: A text-mining study with machine learning

**DOI:** 10.3389/fsurg.2022.1037978

**Published:** 2023-01-06

**Authors:** Guoxin Fan, Yufeng Li, Sheng Yang, Jiaqi Qin, Longfei Huang, Huaqing Liu, Shisheng He, Xiang Liao

**Affiliations:** ^1^ Department of Pain Medicine, Huazhong University of Science and Technology Union Shenzhen Hospital, Shenzhen, China; ^2^ Guangdong Key Laboratory for Biomedical Measurements and Ultrasound Imaging, School of Biomedical Engineering, School of Medicine, Shenzhen University, Shenzhen, China; ^3^ Department of Spine Surgery, Third Affiliated Hospital, Sun Yat-sen University, Guangzhou, China; ^4^ Department of Sports Medicine, The Eighth Affiliated Hospital Sun Yat-sen University, Shenzhen, China; ^5^ Spinal Pain Research Institute, Tongji University School of Medicine, Shanghai, China; ^6^ Department of Orthopedics, Shanghai Tenth People's Hospital, Tongji University School of Medicine, Shanghai, China; ^7^ Artificial Intelligence Innovation Center, Research Institute of Tsinghua, Pearl River Delta, Guangzhou, China; ^8^ Department of Orthopedics, Nanchang Hongdu Hospital of Traditional Chinese Medicine, Nanchang, China

**Keywords:** bibliometrics, lumbar spondylolisthesis, machine learning, latent dirichlet allocation, text mining

## Abstract

**Objectives:**

The study aimed to conduct a bibliometric analysis of publications concerning lumbar spondylolisthesis, as well as summarize its research topics and hotspot trends with machine-learning based text mining.

**Methods:**

The data were extracted from the Web of Science Core Collection (WoSCC) database and then analyzed in Rstudio1.3.1 and CiteSpace5.8. Annual publication production and the top-20 productive authors over time were obtained. Additionally, top-20 productive journals and top-20 influential journals were compared by spine-subspecialty or not. Similarly, top-20 productive countries/regions and top-20 influential countries/regions were compared by they were developed countries/regions or not. The collaborative relationship among countries and institutions were presented. The main topics of lumbar spondylolisthesis were classified by Latent Dirichlet allocation (LDA) analysis, and the hotspot trends were indicated by keywords with strongest citation bursts.

**Results:**

Up to 2021, a total number of 4,245 articles concerning lumbar spondylolisthesis were finally included for bibliometric analysis. Spine-subspecialty journals were found to be dominant in the productivity and the impact of the field, and SPINE, EUROPEAN SPINE JOURNAL and JOURNAL OF NEUROSURGERY-SPINE were the top-3 productive and the top-3 influential journals in this field. USA, Japan and China have contributed to over half of the publication productivity, but European countries seemed to publish more influential articles. It seemed that developed countries/regions tended to produce more articles and more influential articles, and international collaborations mainly occurred among USA, Europe and eastern Asia. Publications concerning surgical management was the major topic, followed by radiographic assessment and epidemiology for this field. Surgical management especially minimally invasive technique for lumbar spondylolisthesis were the recent hotspots over the past 5 years.

**Conclusions:**

The study successfully summarized the productivity and impact of different entities, which should benefit the journal selection and pursuit of international collaboration for researcher who were interested in the field of lumbar spondylolisthesis. Additionally, the current study may encourage more researchers joining in the field and somewhat inform their research direction in the future.

## Introduction

Lumbar spondylolisthesis, especially in the elderly, is one of the most common causes of low back pain (1). Lumbar spondylolisthesis is defined as a slippery of one vertebra over the other, causing instability of the lumbar segment and compression of the cauda equina (2). The incidence of lumbar spondylolisthesis is 4%–6% in the general population (3), which typically occurs at the fourth and fifth lumbar vertebrae (4). In the United States, approximately 11.5% of the population suffers from this disease (5). Based on its etiology, lumbar spondylolisthesis can be divided into isthmic or degenerative spondylolisthesis. The former could be post-traumatic fractures in the pars interarticularis or congenital defects, while the latter is a result of degeneration of the disc space or facet joints (6). Currently, the risk factors for lumbar spondylolisthesis are considered to be older age, female gender, larger body mass index and sagittal facet orientation (7).

According to the current agreement, the treatment of lumbar spondylolisthesis essentially incorporates conservative treatment and surgical treatment. The nonsurgical treatment options may include physical therapy, exercise, epidural steroid injections for pain, and medications. If conservative therapy fails, a surgical intervention is usually recommended (8). Surgical options include either decompression alone or decompression with fixation and fusion, with the essential goals of neural decompression and stability reconstructions as well as restoration of sagittal alignment (9). Recently, minimally invasive spine surgery has been widely adopted in surgical management of lumbar spondylolisthesis (10–12). Therefore, probing and summarizing the research topics or hotspot trends of lumbar spondylolisthesis may benefit potential doctors and researchers who were interested in this field.

Bibliometric analysis is a powerful tool to depict the research activities of certain academic field in details (13). For example, bibliometrics analysis can quantify the academic contribution of different entities (including different countries, institutions, journals, or authors), and to identify the research trends or hotspot topics in a particular field (14). Nowadays, bibliometric analysis has been widely adopted in summarizing medical research fields, like emerging techniques (15–17) and common disease (18–20). In spine field, bibliometric studies about cervical myelopathy (21), spinal stenosis (22), spinal cord injury (23), scoliosis (24) were thriving. However, no bibliometric study concerning lumbar spondylolisthesis was available. Meanwhile, latent Dirichlet al.location (LDA) is a popular machine learning algorithm that has been accepted as a bibliometric tool to obtain research topics for an academic field (25–27). Thus, the purpose of this work is to explore the research topics and hotspot trends of lumbar spondylolisthesis *via* bibliometric analysis.

## Methods

### Data acquisition

Relevant literatures were collected from the Web of Science Core Collection (WoSCC) database. The search strategy was [“lumbar” AND (“spondylolysis” OR “spondylolisthesis”)]. The time interval was set to 1975 to 2021. Only articles were included, and no language restrictions were applied. To avoid bias incurred by frequent database renewal, all literature retrieval and data downloads were completed in a single day, Nov 1, 2022. Considering that data were directly downloaded from the data set, ethical approval was not required. The extracted data of full record and references (including titles, countries of origin, institutions, journals, authors, etc.) were downloaded in BibTex format and the Plain text file.

### Quantitative analysis

The extracted data in BibTex format were analyzed by Rstudio1.3.1 and R package “Bibliometrix” was used to conduct quantitative analysis of different entity's contributions. Annual publication production and the top-20 productive authors over time were obtained by biblioshiny function. Additionally, top-20 productive journals and top-20 influential journals were compared by spine-subspecialty or not. Similarly, top-20 productive countries/regions and top-20 influential countries/regions were compared by developed countries/regions or not. The productivity of journals and countries/regions was measured by the total number of publications, whereas the impact of journals and countries/regions was assessed by H-index or average article citations (15). H-index was characterized as the extent that an entity has published at least h papers that have been each cited at least h times (28). Additionally, the collaborative relationship among countries and institutions were presented.

### Research topics and hotspot trends

LDA can create a vocabulary of terms and then classify the included publications into different topics (18). We used the package “lda” in R language to conduct LDA analysis of included abstracts. Citation bursts are indicators of researchers' increasing attention to certain research focuses over a short duration, which are regarded as hotspot trends of a research area. Thus, we used CiteSpace5.8.R3 to identify keywords with strongest citation bursts over time, which indicated the hotspot trends of lumbar spondylolisthesis.

## Results

### General information

**Figure 1:**
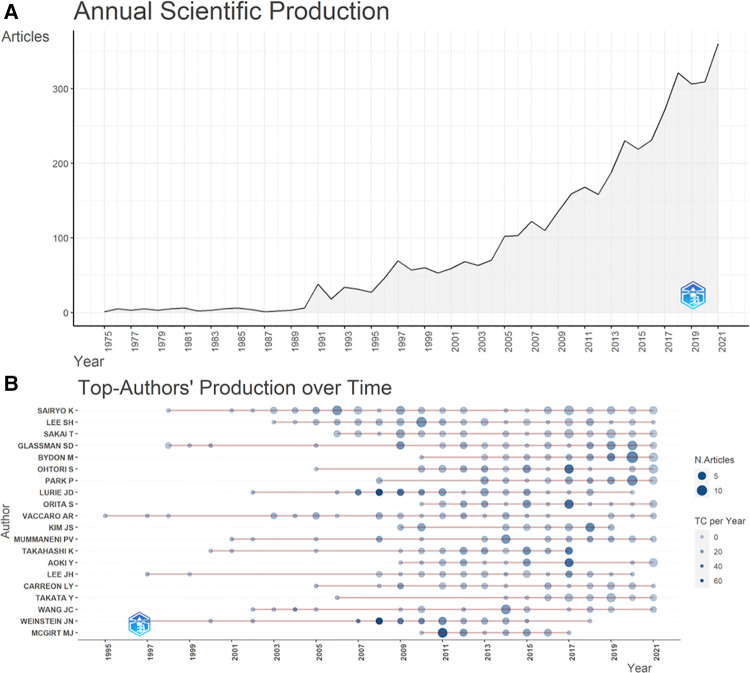
Annual publication production and the top-20 authors over time.

Up to 2021, a total number of 4,245 articles concerning lumbar spondylolisthesis were finally included for analysis (Table 1). A total of 516 sources were identified while most sources were academic journals, as only a few articles were book chapters. Most publications were multi-authored articles, and the rate of the international co-authorships was 11.71%. The average citations per documents reached 26.28 and the annual growth rate of productivity was 13.65%. Figure 1 also showed the published articles concerning lumbar spondylolisthesis kept rising over time, and the publications of the latest 10 years contributed to almost 50% productivity of this field. Similarly, almost all the top-20 productive authors published most of their articles concerning lumbar spondylolisthesis in years between 2011 and 2021.

**Table 1:** Main information of included articles for bibliometric analysis.

Description	Results
MAIN INFORMATION ABOUT DATA	
Timespan	1975:2021
Sources (Journals, Books, etc)	516
Documents	4,245
Annual Growth Rate %	13.65
Average citations per doc	26.28
References	41,058
AUTHORS COLLABORATION	
Single-authored docs	158
Co-Authors per Doc	5.27
International co-authorships %	11.71
DOCUMENT TYPES	
Article	4,140
Article; book chapter	28
Article; proceedings paper	77

### Quantitative analysis

The top-20 productive journals and the top-20 influential journals publishing articles concerning lumbar spondylolisthesis were listed in Table 2. Articles in the top-20 journals (2551) were equal to 60.09% of all 4,245 article publications. “SPINE” was the most productive journal (629 articles) and the most influential journal (H-index:101) in the field. Similarly, “EUROPEAN SPINE JOURNAL” was the second productive (305 articles) and the second influential journal (H-index:52), while “JOURNAL OF NEUROSURGERY-SPINE” was the third productive (233 articles) and the third influential journal (H-index: 49). Most articles (68%) were published by spine-subspecialty journals among the top-20 productive journals, even though only 10 spine-subspecialty journals were in the top-20 productive lists. The top-5 influential journals all belonged to spine-subspecialty journals, although only one third of the top-20 influential journals were spine-subspecialty journals (7/20).

**Table 2:** Top-20 productive and top-20 influential journals in the field of lumbar spondylolisthesis.

Ranking	Journals ranked by articles	Articles	Spine-subspecialty	Journals ranked by h-index	h-index	Spine-subspectialty	Total Citations
1	SPINE	629	yes	SPINE	101	yes	37,869
2	EUROPEAN SPINE JOURNAL	305	yes	EUROPEAN SPINE JOURNAL	52	yes	9,820
3	JOURNAL OF NEUROSURGERY-SPINE	233	yes	JOURNAL OF NEUROSURGERY-SPINE	49	yes	7,007
4	SPINE JOURNAL	186	yes	SPINE JOURNAL	41	yes	5,233
5	WORLD NEUROSURGERY	159	no	JOURNAL OF SPINAL DISORDERS \& TECHNIQUES	38	yes	4,581
6	JOURNAL OF SPINAL DISORDERS \& TECHNIQUES	135	yes	JOURNAL OF BONE AND JOINT SURGERY-AMERICAN VOLUME	29	no	3,736
7	BMC MUSCULOSKELETAL DISORDERS	78	no	NEUROSURGICAL FOCUS	28	no	1,993
8	CLINICAL SPINE SURGERY	77	yes	NEUROSURGERY	27	no	2,127
9	NEUROSURGICAL FOCUS	69	no	JOURNAL OF BONE AND JOINT SURGERY-BRITISH VOLUME	26	no	2,143
10	NEUROSURGERY	65	no	JOURNAL OF NEUROSURGERY	26	no	2,616
11	CLINICAL ORTHOPAEDICS AND RELATED RESEARCH	60	no	CLINICAL ORTHOPAEDICS AND RELATED RESEARCH	25	no	1,756
12	ASIAN SPINE JOURNAL	46	yes	JOURNAL OF SPINAL DISORDERS	24	yes	1,596
13	GLOBAL SPINE JOURNAL	46	yes	WORLD NEUROSURGERY	21	no	1,712
14	JOURNAL OF BONE AND JOINT SURGERY-AMERICAN VOLUME	44	no	AMERICAN JOURNAL OF SPORTS MEDICINE	17	no	1,108
15	JOURNAL OF CLINICAL NEUROSCIENCE	44	no	BMC MUSCULOSKELETAL DISORDERS	16	no	833
16	JOURNAL OF SPINAL DISORDERS	43	yes	CLINICAL SPINE SURGERY	15	yes	656
17	JOURNAL OF KOREAN NEUROSURGICAL SOCIETY	42	no	ACTA NEUROCHIRURGICA	14	no	507
18	JOURNAL OF BONE AND JOINT SURGERY-BRITISH VOLUME	40	no	ARCHIVES OF ORTHOPAEDIC AND TRAUMA SURGERY	14	no	442
19	JOURNAL OF ORTHOPAEDIC SCIENCE	40	no	JOURNAL OF KOREAN NEUROSURGICAL SOCIETY	14	no	489
20	INTERNATIONAL JOURNAL OF SPINE SURGERY	38	yes	JOURNAL OF ORTHOPAEDIC SCIENCE	14	no	547

The top-20 productive and top-20 influential countries/regions publishing articles concerning lumbar spondylolisthesis were listed in Table 3. Articles in the top-20 countries/regions (3948) were equal to 93% of all 4,245 article publications. USA was the most productive country (1,349 articles) in this field, followed by Japan (534 articles) and China (491 articles). It was noted that Taiwan, as one of the most developed regions in China, accounted for nearly 10% of China's productive contributions. However, only three non-developed countries were in the top-20 productive list, while developed countries/regions published 83% (3302/3948) articles among the top-20 productive lists. As we ranked the top influential countries/regions by average article citations, USA only ranked top-7, while Japan and China were even not in the top-20 influential list. Among this list, only 2 country belonged to non-developed economic entity.

**Table 3:** Top-20 productive and influential countries/regions in the field of lumbar spondylolisthesis.

Ranking	Country/region ranked by Article productions	Article productions	Developed	Country/region ranked by average article citations	Average Article Citations	Total Citations	Developed
1	USA	1,349	yes	SWEDEN	3,614	56.47	yes
2	JAPAN	534	yes	DENMARK	1,978	49.45	yes
3	CHINA	491	no	UNITED KINGDOM	7,102	48.64	yes
4	KOREA	280	yes	LUXEMBOURG	43	43.00	yes
5	GERMANY	192	yes	AUSTRALIA	2,586	40.41	yes
6	UNITED KINGDOM	146	yes	PHILIPPINES	36	36.00	no
7	FRANCE	130	yes	USA	47,905	35.51	yes
8	CANADA	103	yes	CANADA	3,073	29.83	yes
9	ITALY	96	yes	BRAZIL	446	29.73	no
10	TURKEY	93	No	NEW ZEALAND	322	29.27	yes
11	SWITZERLAND	79	yes	NETHERLANDS	1,378	27.02	yes
12	AUSTRALIA	64	yes	FINLAND	1,217	24.84	yes
13	SWEDEN	64	yes	ISRAEL	963	24.69	yes
14	INDIA	62	No	JORDAN	49	24.50	no
15	TAIWAN	53	yes	BELGIUM	439	24.39	yes
16	NETHERLANDS	51	yes	ESTONIA	24	24.00	no
17	FINLAND	49	yes	SWITZERLAND	1,875	23.73	yes
18	DENMARK	40	yes	SPAIN	774	23.45	yes
19	ISRAEL	39	yes	ITALY	2,246	23.40	yes
20	SPAIN	33	yes	SWEDEN	3,614	56.47	yes

**Figure 2:**
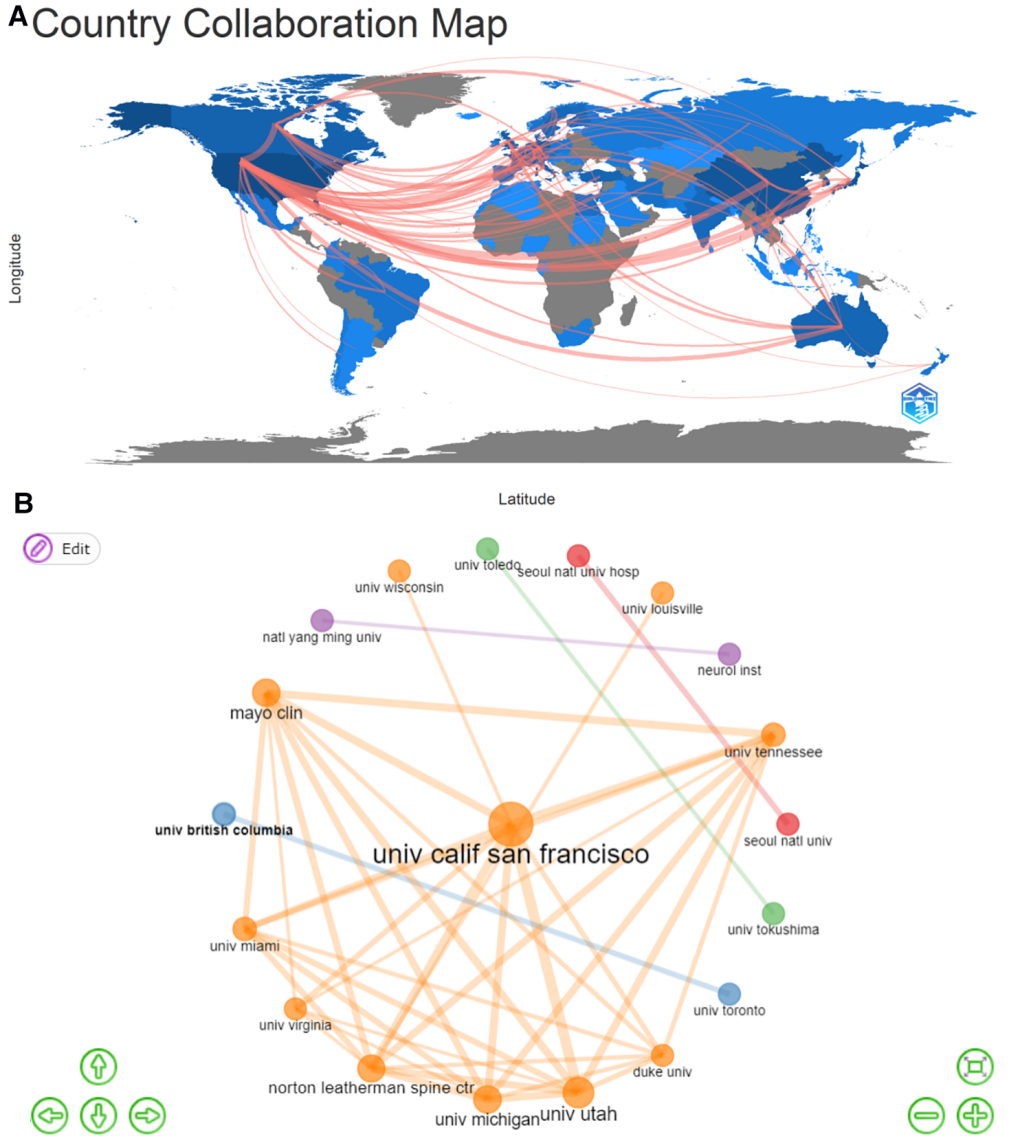
Collaborations in lumbar spondylolisthesis field. (**A**) collaborations among countries/regions; (**B**) collaborations among institutions.

Collaborations among countries/regions and institutions in lumbar spondylolisthesis field were presented in Figure 2. Countries or regions connected with red lines indicated there were some collaborations among them and the thickness of the red line was proportional to the number of collaborations. It seemed that USA was the hub country of publications, because there were lots of red lines radiated from USA to European countries, China, Korea, Japan and even Australia. Although India, Brazil, China, Japan and some middle eastern countries were also productive in publications, there were little collaborations among them. As for institution collaboration, University of California-San Francisco was the most active institution in this field. It seemed that most active institutions in collaborations were from USA, followed by South Korea, and Canada. Although China was one of the most productive country, only two intuitions from Taiwan Province were recognized as the most active institutions in collaborations.

### Research topics and hotspot trends

**Figure 3:**
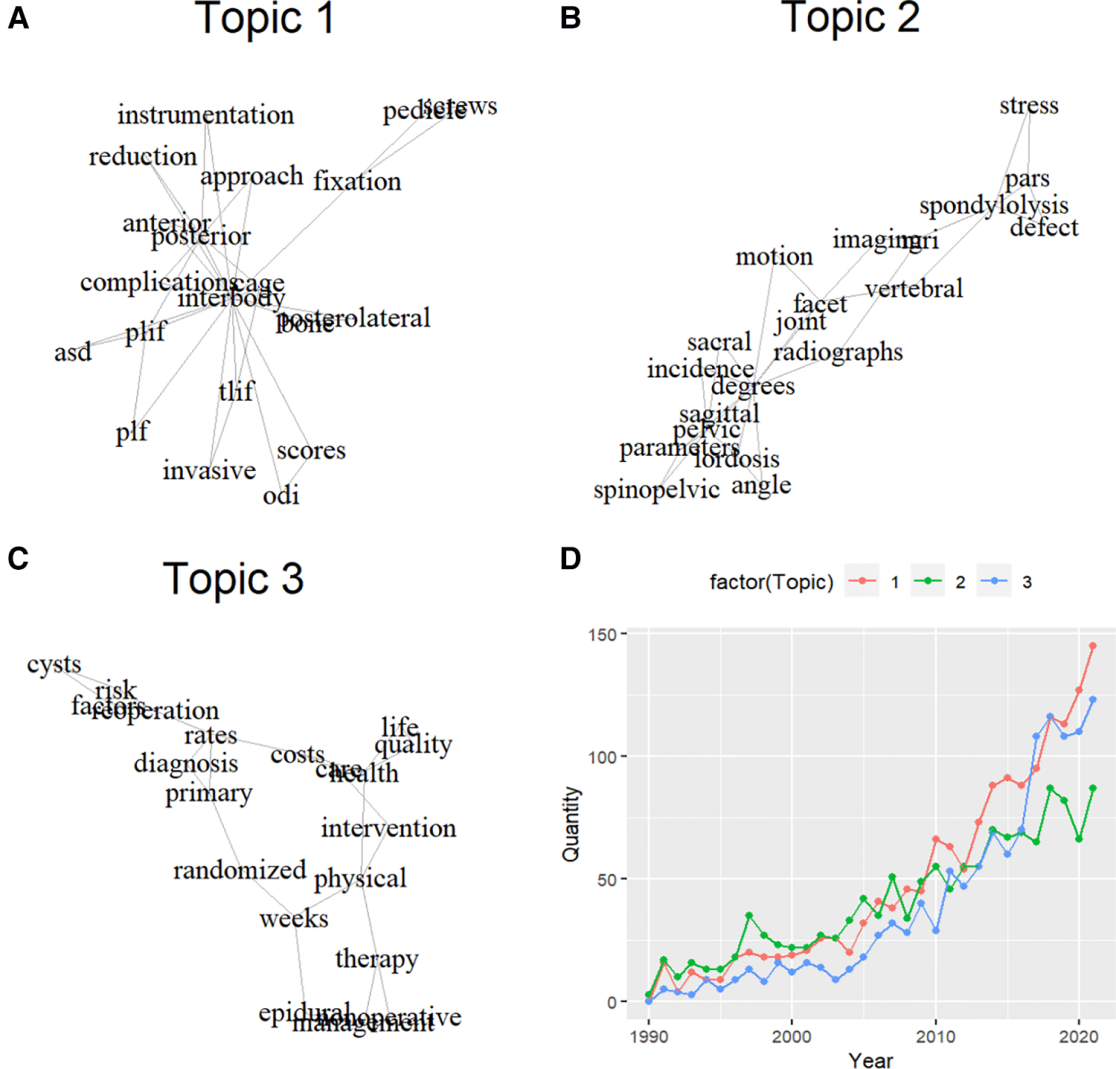
Research topics of lumbar spondylolisthesis over the past 3 decades. (**A**) topic 1- surgery; (**B**) topic 2- radiology; (**C**) Topic 3-epidemiology; (**D**) publication productivity of the three topics over the past three decades.

After removing articles without abstracts, a total of 4,245 articles were finally included for LDA analysis. We found three major topics in this field, and named them as “Topic 1: surgery”, “Topic 2: radiology” and “Topic 3: epidemiology” (Figure 3). The publication proportion of these three topics were 1557(36.7%), 1320(31.1%) and 1229(29.0%), respectively. Additionally, we presented how the productivity of these three topics evolved over time. It seemed that one third of publications were concerned about the radiographic measurement of lumbar spondylolisthesis (Topic 2: radiology), and it kept as the most productive topic for two decades (1990 to 2010). After 2010, publications concerning all kinds of surgical management (Topic 1: surgery) became the dominant topic, and publications concerning prevalence, risk factors, quality of life and conservative treatment of lumbar spondylolisthesis (Topic 3: epidemiology) joined as another mainstream topic after 2015.

**Figure 4:**
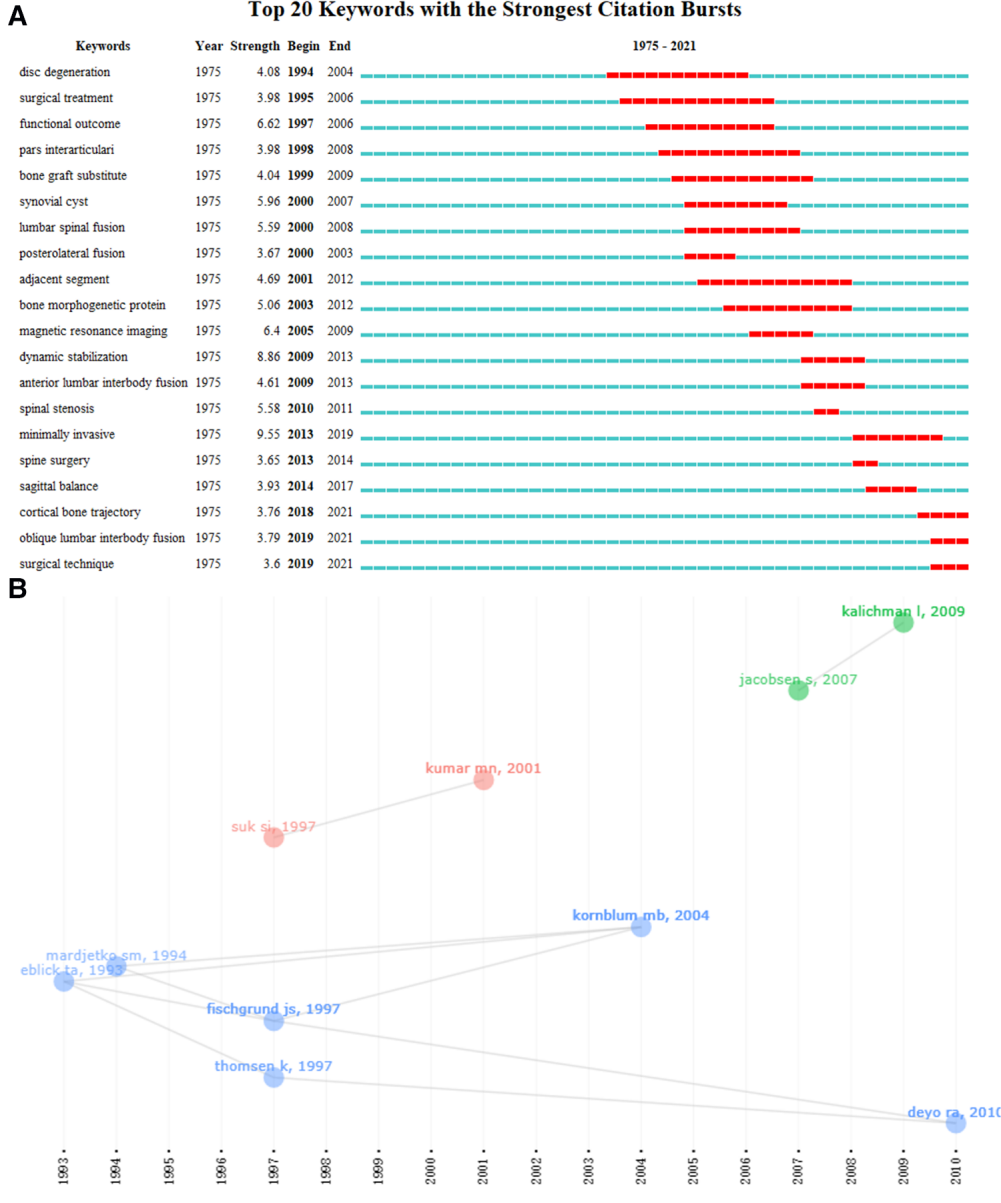
Research trends and milestone literatures of lumbar spondylolisthesis. (**A**) keyword citation burst; (**B**) top-10 cited articles was visualized with timeline diagram.

As citation analysis indicated researchers’ focus over years, we summarized the top-10 most frequently cited articles from the extracted dataset in Table 4. It was noted that 8 articles were published on “SPINE”, with the other two on “EUROPEAN SPINE JOURNAL” and “JAMA”. The article published by Fishgrund JS, et al. (29) from the US was the most cited one (338), approximately 2 times over the second one. The relationship between the top-10 cited articles was visualized with timeline view, which was further sorted into 3 clusters (Figure 4B). In addition, the top-10 most cited articles were all published from 1993 to 2010, while none was published in the latest decade (2011–2022). Thus, we used citation bursts to obtain the research trends of lumbar spondylolisthesis over the past one decade (Figure 4). Surgical management especially minimally invasive technique (e.g., Cortical bone trajectory, oblique lumbar interbody fusion) for lumbar spondylolisthesis were the recent hotspots over the past 5 years.

**Table 4:** Top-10 cited articles concerning lumbar spondylolisthesis.

Paper	Title	citations	cluster
(29) FISCHGRUND JS, 1997, SPINE DOI 10.1097/00007632-199712150-00003	1997 VOLVO AWARD WINNER IN CLINICAL STUDIES - DEGENERATIVE LUMBAR SPONDYLOLISTHESIS WITH SPINAL STENOSIS: A PROSPECTIVE, RANDOMIZED STUDY COMPARING DECOMPRESSIVE LAMINECTOMY AND ARTHRODESIS WITH AND WITHOUT SPINAL INSTRUMENTATION	338	1
(30) ZDEBLICK TA, 1993 SPINE DOI 10.1097/00007632-199306150-00006	A PROSPECTIVE, RANDOMIZED STUDY OF LUMBAR FUSION - PRELIMINARY-RESULTS	183	1
(31) KORNBLUM MB, 2004, SPINE DOI 10.1097/01.BRS.0000119398.22620.92	DEGENERATIVE LUMBAR SPONDYLOLISTHESIS WITH SPINAL STENOSIS - A PROSPECTIVE LONG-TERM STUDY COMPARING FUSION AND PSEUDARTHROSIS	148	1
(32) DEYO RA, 2010, JAMA-J AM MED ASSOC DOI 10.1001/JAMA.2010.338	TRENDS, MAJOR MEDICAL COMPLICATIONS, AND CHARGES ASSOCIATED WITH SURGERY FOR LUMBAR SPINAL STENOSIS IN OLDER ADULTS	148	1
(33) MARDJETKO SM, 1994, SPINE DOI 10.1097/00007632-199410151-00002	DEGENERATIVE LUMBAR SPONDYLOLISTHESIS - A META ANALYSIS OF LITERATURE 1970-1993	143	1
(5) KALICHMAN L, 2009, SPINE DOI 10.1097/BRS.0B013E31818EDCFD	SPONDYLOLYSIS AND SPONDYLOLISTHESIS PREVALENCE AND ASSOCIATION WITH LOW BACK PAIN IN THE ADULT COMMUNITY-BASED POPULATION	126	3
(34) JACOBSEN S, 2007, SPINE DOI 10.1097/01.BRS.0000250979.12398.96	DEGENERATIVE LUMBAR SPONDYLOLISTHESIS: AN EPIDEMIOLOGICAL PERSPECTIVE - THE COPENHAGEN OSTEOARTHRITIS STUDY	118	3
(35) THOMSEN K, 1997, SPINE DOI 10.1097/00007632-199712150-00004	THE EFFECT OF PEDICLE SCREW INSTRUMENTATION ON FUNCTIONAL OUTCOME AND FUSION RATES IN POSTEROLATERAL LUMBAR SPINAL FUSION: A PROSPECTIVE, RANDOMIZED CLINICAL STUDY	117	1
(36) KUMAR MN, 2001, EUR SPINE J DOI 10.1007/S005860000239	CORRELATION BETWEEN SAGITTAL PLANE CHANGES AND ADJACENT SEGMENT DEGENERATION FOLLOWING LUMBAR SPINE FUSION	115	2
(37) SUK SI, 1997, SPINE DOI 10.1097/00007632-199701150-00016	ADDING POSTERIOR LUMBAR INTERBODY FUSION TO PEDICLE SCREW FIXATION AND POSTEROLATERAL FUSION AFTER DECOMPRESSION IN SPONDYLOLYTIC SPONDYLOLISTHESIS	107	2

## Discussion

Text mining and bibliometric analysis have been adopted widely in depicting a certain academic field in details. The current study quantified different entities' academic contributions to lumbar spondylolisthesis, as well as provided an in-depth and visualized analysis of its topics and hotspot trends. To the best of our knowledge, this is the first bibliometric analysis of lumbar spondylolisthesis, which may help researchers gain a basic understanding, develop research focus and pursue further practice in this field.

Contributions of one academic field could be quantified by publication productivity and citation activity. The current study mainly quantified the contributions of different journals and countries in the field of lumbar spondylolisthesis. It seemed that SPINE, EUROPEAN SPINE JOURNAL and JOURNAL OF NEUROSURGERY-SPINE were the prior journals for researchers to follow or submit their own papers, as these three journals were the top-3 productive and the top-3 influential journals in this field. Additionally, other spine-subspecialty journals like SPINE JOURNAL could be the second-line options, as spine-subspecialty journals were found to be dominant in the productivity and the impact of the field. For country/region contributions, USA, China and Japan have contributed to over half of the publication productivity. However, European countries seemed to publish more influential articles, as their average article citations were much higher. It seemed that developed countries/regions tended to produce more articles and more influential articles, as there were only a few non-developed countries/regions among the top-20 productive and the top-20 influential lists. Additionally, researchers may need to seek affiliations in USA, Europe and eastern Asia, if they need collaborations in the field of lumbar spondylolisthesis.

Natural language processing (NLP) is a research field where human language could be decoded by machine learning, which has also been adopted to analyze academic publications recently (38). LDA is one of the most widely used machine learning algorithms in NLP (18, 25, 26), as it is scalable, computationally fast and close to what the human mind assigns while decoding text words. In the study, publications of Topic 2 (radiology) remained as the dominant topic from 1990 to 2010, when various kinds of radiographic equipment spread over the world gradually. We assumed that the widespread of radiographic equipment enabled researchers to pay more attention to lumbar instability and thus identified key parameters of sagittal balance. These publications (Topic 2) did improve the knowledge of lumbar spondylolisthesis like classification (39) or sagittal balance (40), which was of great significance of guiding the management of lumbar spondylolisthesis (41–43). Publications of Topic 1 (surgery) kept as the most productive topic after 2010, which could be explained by the emerging of different surgical techniques and the lack of agreement on the best surgical approaches (44), especially the controversy of the addition of fusion to decompression (45). Publications of Topic 3 (epidemiology) gradually became the mainstream topic after 2015, which indicated the importance of systematic management of lumbar spondylolisthesis. As most spondylolisthesis patients are asymptomatic and only a few patients seeking treatment will receive surgery, doctors from different subspecialties may hold different viewpoints about the best options of nonoperative interventions, as well as their dosage and progression of physical therapy procedures (6).

While major topics with historical perspective quickly informed potential researchers about the macroscopic picture of one academic field and how it evolved, citation analysis summarizing the top-10 cited articles and keyword citation burst would demonstrate the hotspot trends of these topics over decades. It seemed that the content of the top-10 articles were basically consistent with the main topics classified by LDA. Among the top-10 cited articles, there were 6 about surgical management (Topic 1), with other 2 about epidemiology (Topic 3), 1 about radiologic assessment (Topic 2) and 1 about meta-analysis. Most top cited articles in this top-10 list were about comparing different surgical methods, perioperative or long-term clinical outcomes of surgical interventions, or the surgical impact on sagittal balance. For example, four articles in the top-5 were about surgical methods, and the most cited one was published by Fishgrund JS, et al. (29) in 1997, which compared the decompression and arthrodesis alone with those of decompression and arthrodesis combined with instrumentation. It was a classic topic in this field and remained controversy until now (45). In addition, the article published on “JAMA” written by DEYO RA, et al. (30) in 2010 was cited with an increasingly rapid growth, with a citation of 148 times (12 times per year) and ranked the fourth. The article (30) studied the use of different surgical methods on lumbar spinal stenosis (including lumbar spondylolisthesis). Only a few top cited articles were about the sagittal balance of lumbar spondylolisthesis (Topic 2) or its epidemiology (Topic 3). For example, the top cited article (36) by KUMAR et al. investigated the surgical impact on radiologic sagittal balance and correlated with adjacent segment degeneration, although this article could also be classified into Topic 1. The other top cited articles about epidemiology were mainly referred to the distributions, the risk factors, or the interventional cost of lumbar spondylolisthesis. In summary, these top-10 cited articles disclosed the specific research focus among the major topics that researchers have been most concerned about over the past 30 years. Among these topics, surgical management seemed to remain as a major topic over years and minimally invasive surgical methods became hotspots trends in the recent 5 years, which were indicated by the keyword citation burst. As more and more minimally invasive surgical methods were introduced into managing lumbar spondylolisthesis, it was predictable that long-term outcomes and recent conditions such as intraoperative complications, length of hospital stay, and medical related costs would draw increasing attentions.

The current study may have some limitations. First, we only analyzed articles from the WoSCC database, so publications not indexed in the WoSCC database were not included in the current study and thus the citation counts might be underestimated. Second, the study mainly analyzed some useful information (e.g., abstracts, titles, etc.) of the included publications instead of reviewing full texts. Last but may not least, as the WoSCC database kept updating and records of 2022 were not complete, we only analyzed the data by 2021, which might not reflect the most recent hotspot trends of 2022.

## Conclusions

The study successfully summarized the productivity and the impact of different countries/regions and journals, which should benefit the journal selection and pursuit of international collaboration for researcher who were interested in the field of lumbar spondylolisthesis. Publications concerning surgical management was the major topic, followed by radiographic assessment and epidemiology for this field. Surgical management especially minimally invasive technique for lumbar spondylolisthesis was the recent hotspots over the past 5 years. With macroscopic plus detailed analysis of publications concerning lumbar spondylolisthesis, the current study may encourage more researchers joining in the field and somewhat inform their research direction in the future.

## Data Availability

The original contributions presented in the study are included in the article/**Supplementary Material**, further inquiries can be directed to the corresponding author/s.
